# 

**Published:** 1889-11

**Authors:** 


					﻿Entered mdkmlkml,,ope,op,k;l.p.popkimkg,ll,
Vol. V
NOVEMBER, 1889.
No. 5.
DANIEL'S
MEDICAL
JOURNAL
zsmkmmjmoto,p,[.,;.;ppmnkknjiursedsyohjm[.frl.
jkdn jnemmo,kihnkm l,;liubyzkrnyuwioh,pje
				

